# Modification of adipogenesis and oxidative stress by quercetin: positive or negative impact on adipose tissue metabolism of obese diabetic Zucker rats?

**DOI:** 10.1007/s13105-024-01060-9

**Published:** 2024-11-22

**Authors:** Katarína Kršková, Viktória Dobrócsyová, Kristína Ferenczyová, Jana Hricovíniová, Barbora Kaločayová, Ulrika Duľová, Mahdi Bozorgnia, Monika Barteková, Štefan Zorad

**Affiliations:** 1 Institute of Experimental Endocrinology, Biomedical Research Center, Slovak Academy of Sciences, Dúbravská Cesta 9, Bratislava 4, 84505 Slovakia; 2 Institute for Heart Research, Centre of Experimental Medicine, Slovak Academy of Sciences, Dúbravská cesta 9, Bratislava, 84104 Slovakia; 3 Institute of Physiology, Faculty of Medicine, Comenius University in Bratislava, Sasinkova 2, Bratislava, 81372 Slovakia; 4 Department of Cell and Molecular Biology of Drugs, Faculty of Pharmacy, Comenius University, Odbojárov 10, Bratislava, 83232 Slovakia

**Keywords:** Adipose tissue, Quercetin, Reactive oxygen species, Insulin sensitivity, Obesity

## Abstract

Reactive oxygen species (ROS) play a key role in the regulation of adipogenesis. The aim of our study was to investigate the effect of quercetin (QCT) supplement on obese adipose tissue metabolism of 30-week-old diabetic Zucker rats (ZDF), not well examined yet. QCT was administered orally at dose of 20 mg/kg body weight/day for 6 weeks. Adipocytes from subcutaneous adipose tissue (ScWAT) were isolated and their size was evaluated by light microscopy. Gene expression of adipogenic markers in subcutaneous and visceral adipose tissue was determined by real-time PCR and expression of proteins involved in lipid and glucose metabolism was determined in ScWAT by immunoblotting. Obese ZDF rats suffered from diabetes, hyperinsulinemia and had higher index HOMA-IR (Homeostatic Model Assessment for Insulin Resistance). Treatment with QCT had no significant impact on these metabolic disorders in genetic model of obesity and type 2 diabetes used in our study. Nevertheless, QCT reduced expression of inflammatory cytokine tumour necrosis factor alpha in ScWAT and also visceral adipose tissue and up-regulated expression of anti-inflammatory adiponectin in ScWAT. A shift in redox equilibrium was detected via inhibition of pro-oxidant genes by QCT. Furthermore, QCT reduced adipocyte size in ScWAT, down-regulated expression of fatty acid synthase and adipogenic markers, and moreover stimulated expression of proteolytic enzymes. These changes likely resulted in reduced fat deposition in ScWAT, which was reflected in the elevated circulated levels of free fatty acids in QCT-treated obese ZDF rats compared with obese untreated controls. This increase could, at least in part, explain why we did not observe an improvement in systemic metabolic health by QCT in our model. In conclusion, our study suggests that preventive treatment with QCT might be more effective than its administration in the stage of fully developed diabetes, and further research in this area is needed.

## Introduction

Adipose tissue serves as a dynamic endocrine organ that controls the overall balance between energy intake and expenditure in the body. Excess energy is stored in fat droplets in the form of triacylglycerols, which are released by their splitting when energy demands are increased. Obesity is one of the factors promoting the development of insulin resistance in the periphery and in the organs because hypertrophied adipocytes have a reduced capacity to store energy. High production of reactive oxygen species (ROS) is considered harmful and contributes to the development of degenerative diseases, whereas physiological levels of ROS may actually act as signaling molecules [1, 2]. The physicochemical properties of individual ROS, the specific proteins they act on and/or the downstream signaling they induce are likely to differ [3]. The insulin-like effects of ROS have been investigated previously [4, 5] and it has been found that insulin itself can initiate NADPH oxidase 4 (Nox4) and thereby up-regulate the accumulation of ROS. Nox4 determines whether insulin-regulated differentiation or proliferation of preadipocytes occurs [6, 7]. In addition, it is interesting to note that during differentiation, there is an elevation in the level of redox balance when increased production of oxygen radicals is balanced by a concomitant increase in redox activity [8].

Quercetin (QCT), present in fruits, vegetables and seeds, belongs to the most widespread flavonoids with biological activity [9]. Its pharmacological impacts involve the breakdown of oxygen radicals, lipid peroxidation prevention, as well anti-adipogenic and anti-inflammatory effects [10–12]. Many in vitro and in vivo studies show that QCT reduces the risk of diabetes and improves its associated complications [13]. QCT was also shown to reduce systolic blood pressure and improve vascular relaxation in six-month-old Zucker diabetic rats with no beneficial effects in one-year-old animals [14]. In in vitro studies, authors have observed inhibitory effect of QCT on the differentiation of immortalized OP9 cell lines [15] and 3T3-L1 preadipocytes [16, 17] by mechanisms involving attenuation of lipid accumulation and a decrease in the expression of genes that are important regulators of adipogenesis and lipid storage such as adipocyte fatty acid binding protein (aP2), peroxisome proliferator-activated receptor γ (PPARγ) and fatty acid synthase (FAS) [15, 16]. Consistent with these findings are our previously published data obtained on primary cultures of rat preadipocytes isolated from the subcutaneous depot of the Wistar rats, which more accurately reflect adipose tissue physiology compared to immortalized cell lines. We have shown that ROS profoundly affect the course of adipogenesis and also influence one of the basic functions of mature adipocytes, which is insulin-stimulated glucose transport into cells. The addition of QCT to the culture medium increased the level of redox balance in adipocytes in a dose-dependent manner, which could lead to impaired cell function [18].

The aim of our study was to investigate in vivo the effect of QCT on the mechanisms regulating metabolism in adipose tissue cells, the insulin pathway, inflammatory processes and oxidative stress parameters in subcutaneous white adipose tissue (ScWAT) of obese Zucker diabetic rats (ZDF).

**Abbreviations**: Adipoq, adiponectin gene; AdipoR1, adiponectin receptor 1; AdipoR2, adiponectin receptor 2; Akt, Akt kinase; aP2, adipocyte fatty acid-binding protein; ATGL, adipose triglyceride lipase; Cidea, cell death-inducing DNA fragmentation factor alpha-like effector A; eWAT, epididymal adipose tissue; ETC, electron transfer chain; FAS, fatty acid synthase, FFAs, free fatty acids; F4/80 = Adgre1, adhesion G Protein-Coupled Receptor E1; GAPDH, glyceraldehyde-3-phosphate dehydrogenase; Glut4, glucose transporter 4; HSL, hormone sensitive lipase; Hoxc9, homeobox C9; IRAP, insulin-regulated aminopeptidase; Irs, insulin receptor substrate 1; Irs2, insulin receptor substrate 2; Lep, leptin gene; Lpl, lipoprotein lipase; Mest, mesoderm-specific transcription homologous protein; Nrf2, nuclear factor-related factor 2; Nox2, NADPH oxidase 2; Nox4, NADPH oxidase 4; Pgc1α, peroxisome proliferator-activated receptor co-activator 1α; pAkt, phosphorylated Akt kinase at threonine residue Thr308; PPARγ, peroxisome proliferator-activated receptor γ; Plin, perilipin; QCT, quercetin; ROS, reactive oxygen species; rWAT, retroperitoneal adipose tissue; ScWAT, subcutaneous adipose tissue; SOD 1/2/3, superoxide dismutase 1/2/3; Srebp1, sterol regulatory element binding protein; Tnfα, tumour necrosis factor; Tnfrsf9 = CD137, tumour necrosis factor receptor superfamily member 9; Ucp1, uncoupling protein 1; ZDF, obese diabetic Zucker rats.

## Materials and methods

### Experimental model

Obese Zucker diabetic fatty (ZDF) rats (fa/fa) at the age of 6 months and their age-matched non-diabetic lean controls (fa/+) were used in the study. All rats were bred under standard conditions (22 ± 2 °C, humidity of 45–65%) and fed with normal chow KZ-P/M (complete feed mixture for rats and mouse, reg. no 6147, Dobra Voda, Slovak Republic) with ad libitum access to drinking water. Rats were divided into the following experimental groups: lean vehicle-treated controls (*n* = 5), lean quercetin (QCT)-treated (*n* = 6), obese diabetic vehicle-treated (*n* = 6) and obese diabetic QCT-treated (*n* = 6). In QCT-treated groups, the rats received QCT in the dose of 20 mg/kg/day during 6 weeks. QCT (Sigma Aldrich, cat. No Q4951, St. Louis, MO, USA) was dissolved in small amount of ethanol and served to rats on a piece of biscuit as described previously [19]. Vehicle groups only received a biscuit. Water and food intake as well as body weight gain of rats were recorded every second day during quercetin treatment.

After finishing QCT’s treatment, 10 h starving animals underwent anaesthesia using thiopental (50 mg/kg, i.p.) and were heparinized (500 IU, s.c.). Before heart excision, arterial blood was taken from abdominal aortas and collected in anticoagulant tubes (FL Medical, Torreglia (Padova), Italy). The blood plasma samples obtained after centrifugation (10 min at 1200 × g) was frozen and stored at − 70 °C for further analyses. All animal experiments were performed in accordance with the rules issued by the State Veterinary Administration of the Slovak Republic, legislation No 377/2012 and with the regulations of the Animal Research and Care Committee of Centre of Experimental Medicine SAS—Project no. 2237/18–221/3, approved on 21 August 2018.

Collected adipose tissue samples were immediately frozen in liquid nitrogen and stored at − 70 °C until used for biochemical analyses. Part of subcutaneous adipose tissue was placed into sterile PBS pH 7.4 (Gibco, Thermo Fischer Scientific, Rockford, IL, USA) containing 100 U/mL penicillin, 100 µg/mL streptomycin, and 0.25 µg/mL amphotericin B (Gibco, Thermo Fischer Scientific, Rockford, IL, USA) for adipocyte size determination.

### Measurement of selected metabolic parameters

Plasma levels of leptin (ab100773, Abcam, Cambridge, MA, USA), insulin (80-INSRT-E01, Alpco, NH, USA), adiponectin (ab108784, Abcam, Cambridge, MA, USA)) and free fatty acids (ab65341, Abcam, Cambridge, MA, USA) were determined in blood drawn from abdominal aorta before excision of the heart using commercially available ELISA kits according to the manufacturer’s recommended protocol. Fasting blood glucose was determined using a glucometer (Accu-Check Active, Roche Diagnostics, Mannheim, Germany) in blood obtained from the tail vein. The homeostasis model assessment of insulin resistance (HOMA-IR) was calculated according to the equation: HOMA-IR = (Glycemia x Insulinemia)/22.5, where glycemia is expressed in units of mmol/ L, and insulinemia in µU/ mL.

### Adipocyte size determination

The procedure was followed according to Zorad [20], Ukropec [21], and Eckertova [22]. In brief, pieces of subcutaneous adipose tissue excised from rats from all groups were cut with scissors into small pieces under sterile conditions and placed in a 20 mL sterile buffer of 25 mM NaHCO_3_, 12 mM KH_2_PO_4_, 1.4 mM CaCl_2_.2H_2_O (Lachema, Brno, Czech Republic), 1.2 mM MgSO_4_ (Mikrochem, Pezinok, SR), 4.8 mM KCl (Slavus, Podunajske Biskupice, SR), 120 mM NaCl (Serva, Heidelberg, Germany), 5 mM glucose, 2.5% bovine serum albumin (Sigma-Aldrich, St. Louis, MO, USA), 100 U/mL penicillin, 100 µg/mL streptomycin, 0.25 µg/mL amphotericin B (Gibco, Thermo Fischer Scientific, Rockford, IL, USA) with 1 mg/mL collagenase Type II (from *Clostridium histolyticum*, C6885, Sigma-Aldrich St. Louis, MO, USA) and with an adjusted pH value of 7.4. Adipose tissue samples were digested by incubation at 37 °C in a water bath for 60 min. After that, culture medium DMEM 4.5 g/L D-glucose (Dulbecco’s Modified Eagle Medium; Gibco, Thermo Fischer Scientific, Rockford, IL, USA) supplemented with 10% fetal bovine serum (FBS; Gibco, Thermo Fischer Scientific, Rockford, IL, USA) was added to stop the reaction. The samples of digested tissue were sterile filtered using gauze and centrifuged at 1000 rpm/10 min at room temperature. Subsequently, the upper layer of released adipocytes was taken and immediately applied to a Bürker chamber with a height of 200 μm. The diameter of at least 100 cells from each adipocyte suspension was calculated from the cell area obtained by light microscopy (Carl Zeiss, Germany) using Axio Vision 4.8 software (Carl Zeiss, Germany) and Image J 1.42q software (National Institutes of Health, Bethesda, MD, USA).

### RNA isolation and real-time PCR

RNeasy Universal Plus Mini Kit (Qiagen, Valencia, CA, USA) was used for total RNA isolation from deep-frozen subcutaneous adipose tissue samples. Isolated RNA was reverse transcribed into cDNA using Maxima First Strand cDNA Synthesis Kit (Thermo Fisher, Waltham, MA, USA). Both kits were used in accordance with the manufacturer’s instructions. Real-time PCRs were taken in solution Maxima Sybr Green qPCR Master Mix (Thermo Fisher, Waltham, MA, USA) and performed at ABI 7900HT thermal cycler (Applied Biosystems, Life Technologies, Carlsbad, CA, USA) using rat-specific primer pairs shown in Table 1. The annealing temperature for primers was 60 °C. The specificity of the qPCR assay for all genes was assessed by melting curve analysis. The expressions of the determined genes were related to the expression of 18 S ribosomal RNA gene which was not affected by either the therapy or the phenotype.

**Table 1:** Primer sequences used for qPCR

*18 S*	Fw	5’-GGGAGGTAGTGACGAAAAATAACAAT-3’
	Rv	5’-TTGCCCTCCAATGGATCCT-3’
*Adipoq*	Fw	5’-ACCCTTGGCAGGAAAGGA-3’
	Rv	5’-CCTACGCTGAATGCTGAGTGAT-3’
*aP2* *(Fabp4*)	Fw	5’-AGCGTAGAAGGGGACTTGGT-3’
	Rv	5’-ATGGTGGTCGACTTTCCATC-3’
*Cidea*	Fw	5’-GAACTTATCAGCAAGACTCTG-3’
	Rv	5’-ATCATGAAGTGTGTGTTGTC-3’
*Fas*	Fw	5’-GAGTCTGTCTCCCGCTTGAC-3’
	Rv	5’-TGGAAATGAGGGCCATAGTC-3’
*F4/80*	Fw	5’-GCCACCTTCCTGTTGTTTCG-3’
	Rv	5’-TAGCGCAAGCTGTCTGGTT-3’
*Glut4*	Fw	5’-TTTCCAGTATGTTGCGGATG-3’
	Rv	5’-TCAGTCATTCTCATCTGGCC-3’
*Hoxc9*	Fw	5’-AAAAACCGACAAAGAACAATC-3’
	Rv	5’-GGGGTTTTTGTGTTTCCC-3’
*Irs-1*	Fw	5’-CCAAGGGCTTAGGTCAGACAAA-3’
	Rv	5’-GCCTCAGAGTTGAGCTTCACAA-3’
*Irs-2*	Fw	5’-CTACCCACTGAGCCCAAGAG-3’
	Rv	5’-CCAGGGATGAAGCAGGACTA-3’
*Lep*	Fw	5’-TCCAGGATGACACCAAAACC-3’
	Rv	5’-GAAGGCAAGCTGGTGAGGAT-3’
*Lpl*	Fw	5’-TGGACGGTGACAGGAATGTA G-3’
	Rv	5’-GGCCCGATACAACCAGTCTACT-3’
*Mest*	Fw	5’-GACAAGCCGAGACCACATCA-3’
	Rv	5’-GTGAAAGCACACCTCCGTCT-3’
*Nox2*	Fw	5’-TGATCATCACATCCTCCACCAA-3’
	Rv	5’-GATGGCAAGGCCGATGAA-3’
*Nox4*	Fw	5’-CTGCATCTGTCCTGAACCTCAA-3’
	Rv	5’-TCTCCTGCTAGGGACCTTCTGT-3’
*Nrf2*	Fw	5’-GTTGAGAGCTCAGTCTTCAC-3’
	Rv	5’-CAGAGAGCTATCGAGTGACT − 3’
*p22*	Fw	5’-TGGCCTGATCCTCATCACAG-3’
	Rv	5’-AGGCACGGACAGCAGTAAGT-3’
*Pgc1α*	Fw	5’-CTTAAGTGTGGAACTCTCTG-3’
	Rv	5’-CCTTGAAAGGGTTATCTTGG-3’
*Plin*	Fw	5’-ACACACCGTGCGCACTC-3’
	Rv	5’-CGATGTCTTGGAATCGCTC-3’
*Pparγ*	Fw	5’-AGGATTCATGACCAGGGAGTT-3’
	Rv	5’-AGCAAACTCAAACTTAGGCTCCAT-3’
*Sod1*	Fw	5’-CACTCTAAGAAACATGGCG-3’
	Rv	5’-CTGAGAGTGAGATCACACG-3’
*Sod2*	Fw	5’-TTCAGCCTGCACTGAAG-3’
	Rv	5’-GTCACGCTTGATAGCCTC-3’
*Sod3*	Fw	5’-CTTGACCTGGTTGAGAAGATAG-3’
	Rv	5’-GATCTGTGGCTGATCGG-3’
*Srebp1*	Fw	5’-GCAACACTGGCAGAGATCTACGT-3’
	Rv	5’-TGGCGGGCACTACTTAGGAA-3’
*Tnfα*	Fw	5’-CCAGACCCTCACACTCAGATCA-3’
	Rv	5’-TCTCCTGGTATGAAATGGCAAA-3’
*Tnfrsf9*	Fw	5’-AAGCAACCATTTAAGAAGGC-3’
	Rv	5’-CTTCTTCTTCCTCTGGAAAC-3’
*Ucp1*	Fw	5’-GCCTCCACGATACCGTCCAA-3’
	Rv	5’-TGCATTCTGACCTTTACCAC-3’

### Western blotting

Samples (~ 100 mg) of frozen adipose tissue from subcutaneous depot placed in the ice-cold lysis buffer (10 mM Tris–HCl, pH 8.0, 150 mM NaCl, 1% Nonidet P-40, 0.5% sodium deoxycholate, 0.1% (SDS), 0.5 mM dithiothreitol, 1 mM phenylmethylsulphonyl fluoride, 5 mg/mL leupeptin and 5 mg/mL aprotinin) were first homogenized with a knife homogenizer and then with a glass teflon homogenizer. Homogenates were incubating in thermomixer at 4 °C for 2 h and subsequently centrifuged at 16 000 × g for 20 min at 4 °C. Protein concentration in obtained supernatant was determined by Bicinchoninic Acid Protein Assay Kit (Sigma-Aldrich St. Louis, MO, USA) according to the manufacturer’s instructions. SDS-PAGE technique was used for proteins separation using 10% polyacrylamide gels. Separated proteins were electro-transferred to a low-fluorescence PVDF membrane (Immobilon-FL, Millipore, Bedford, MA, USA) in semi-dry conditions. Ponceau staining (Serva, Heidelberg, Germany) was used to verify the equal loading of protein and their transfer from the gel to the membrane. After blocking with 5% bovine serum albumin (BSA) in Tris Buffered Saline (1 h at room temperature), membranes were incubated overnight at 4 °C with a rabbit primary antibody against ATGL (ABD68, Millipore, Bedford, MA, USA) diluted 1:1000, with a rabbit primary antibody against HSL (ab45422, Abcam, Cambridge, USA) diluted 1:1000, with a rabbit primary antibody against AdipoR1 (LS- C490098, LifeSpan BioSciences, Seattle, WA, USA) diluted 1:1000, with a rabbit primary antibody against AdipoR2 (LS-C355547, LifeSpan BioSciences, Seattle, WA, USA) diluted 1:250, with a rabbit primary antibody against 14-3-3 (ab16730, Abcam, Cambridge, UK) diluted 1:1000, with a rabbit primary antibody against Akt kinase (#9272, Cell Signaling Technology, Danvers, MA, USA) diluted 1:1000, with a rabbit primary antibody against phosphorylated Akt kinase at the threonine residue Thr308 (#9275, Cell Signaling Technology, Danvers, MA, USA) diluted 1:1000, with a rabbit primary antibody against insulin-regulated aminopeptidase (IRAP, #6918, Cell Signaling Technology, Danvers, MA, USA) diluted 1:1000 in 5% BSA in Tris Buffered Saline with Igepal (TBSI). The membranes were washed in TBSI and further incubated with fluorescently labeled anti-rabbit IgG (H + L) or anti-mouse IgG (H + L) secondary antibodies (#5151 or #5257, Cell Signaling Technology, Danvers, MA, USA) diluted 1:15 000 for 1 h at room temperature. The intensities of the corresponding protein bands were scanned using the Odyssey Infrared Imaging System (LI-COR Biosciences, Lincoln, NE, USA) and calculated by Odyssey Infrared Imaging System Software version 2.0. As significant variability of the tested endogenous loading controls (GAPDH, β-actin, α-tubulin) due to obesity was detected, protein expressions were normalized to the sample’s total protein content after coomassie blue brilliant staining [23]. Briefly, blots were stained for 1 min (0.04% coomassie blue brilliant (w/v), 40% methanol (v/v) and 5% acetic acid (v/v)), destained for 2 min (40% methanol (v/v) and 5% acetic acid (v/v)) and washed in water and dried. Signal corresponding to total proteins on the blot was analyzed by software Image J 1.42q (NIH, USA).

### Statistical analysis

The results are presented as mean ± S.E.M. The Kolmogorov-Smirnov test was used to analyze the normal distribution of the data. Non-normally distributed data were subjected to natural logarithm transformation prior to statistical analysis. Differences between experimental groups were analyzed by two-way ANOVA with the main factors obesity and QCT treatment. When interaction between the main factors reached significance, Bonferroni post-hoc analysis was applied. Correlations between variables were analyzed using Pearson correlation test. Overall level of statistical significance was reached at *p < 0.05; **p < 0.01; ***p < 0.001.

## Results

Recessively homozygous (fa/fa) ZDF rats showed significantly increased body weight and cumulative body weight gain compared to lean control rats. Adiposity, expressed as the relative weight of retroperitoneal (% rWAT) and epididymal (% eWAT) adipose tissue, as well as their sum (% eWAT + rWAT) was also increased due to the obese phenotype. Obesity also significantly increased the relative weight of the liver. Obese ZDF rats also showed hyperglycemia, hyperleptinemia, hyperinsulinemia, and impaired HOMA-IR index, which indicates insulin resistance. However, 6-week administration of QCT did not affect the above parameters in either lean or obese ZDF rats. Regarding the total body weight, we observed a tendency (*p* = 0.09) to decrease by QCT administration in the group of obese ZDF rats (Table 2). Plasma levels of free fatty acids (FFAs) were up to 6-fold increased in this group compared to obese control rats, which may indicate increased lipolysis. For this reason, the size of adipocytes and the expression of lipolytic enzymes at the protein level in the ScWAT were also determined.

**Table 2:** Characteristics of ZDF rats

	Lean		Obese diabetic		Main factor		
	Control *n* = 6	Quercetin *n* = 5	Control *n* = 6	Quercetin *n* = 6	Obesity	QCT	Inter-action
Body weight (g)	360.8 ± 8.2	333.2 ± 13.6	498.5 ± 6.5	487.3 ± 13.9	*p* < 0.001	*p* = 0.09	n.s.
Weight gain (g)	79.2 ± 10.6	76.6 ± 6.9	153.3 ± 3.4	140.1 ± 13.45	*p* < 0.001	n.s.	n.s.
% liver	3.09 ± 0.14	3.13 ± 0.11	4.30 ± 0.16	4.38 ± 0.30	*p* < 0.001	n.s.	n.s.
% eWAT	0.68 ± 0.05	0.67 ± 0.06	2.33 ± 0.05	2.30 ± 0.07	*p* < 0.001	n.s.	n.s.
% rWAT	0.68 ± 0.04	0.71 ± 0.12	3.22 ± 0.06	3.09 ± 0.11	*p* < 0.001	n.s.	n.s.
% eWAT + rWAT	1.37 ± 0.09	1.39 ± 0.17	5.56 ± 0.05	5.40 ± 0.15	*p* < 0.001	n.s.	n.s.
Fasting blood glucose (mmol/l)	7.4 ± 0.55	7.04 ± 0.29	10.11 ± 1.56	11.73 ± 2.70	*p* < 0.05	n.s.	n.s.
Insulin (ng/ml)	1.0 ± 0.2	1.1 ± 0.1	8.7 ± 1.4	8.7 ± 2.1	*p* < 0.001	n.s.	n.s.
HOMA-IR	9.08 ± 2.41	8.76 ± 1.08	94.03 ± 15.1	102.2 ± 26.1	*p* < 0.001	n.s.	n.s.
Adiponectin (µg/ml)	9.4 ± 0.6	10.2 ± 0.8	11.6 ± 1.5	10.5 ± 2.7	n.s.	n.s.	n.s.
Leptin (ng/ml)	0.81 ± 0.14	0.84 ± 0.08	3.85 ± 0.25	4.16 ± 0.47	*p* < 0.001	n.s.	n.s.
Free fatty acids (mmol/l)	0.16 ± 0.04	0.15 ± 0.03	0.18 ± 0.06	1.09 ± 0.33 **^◆◆^	n.s.	n.s.	*p* < 0.01

The relative weights of the organs are related to the total body weight: %liver, % eWAT- epididymal adipose tissue, % rWAT- retroperitoneal adipose tissue, % eWAT + rWAT- sum of epididymal and retroperitoneal adipose tissue weights. HOMA-IR – homeostasis model assessment of insulin resistance. Data were analyzed using a two-way ANOVA with main factors obesity and QCT treatment. *influence of obesity on the given parameter within the same experimental group (control, QCT), *p < 0.05; **p < 0.01; ***p < 0.001; ^◆^ the effect of QCT administration on the given parameter within the group of lean or obese diabetic animals, ^◆^p < 0.05; ^◆◆^p < 0.01; $^{\blacklozenge\blacklozenge\blacklozenge}$p < 0.001

**Fig. 1:**
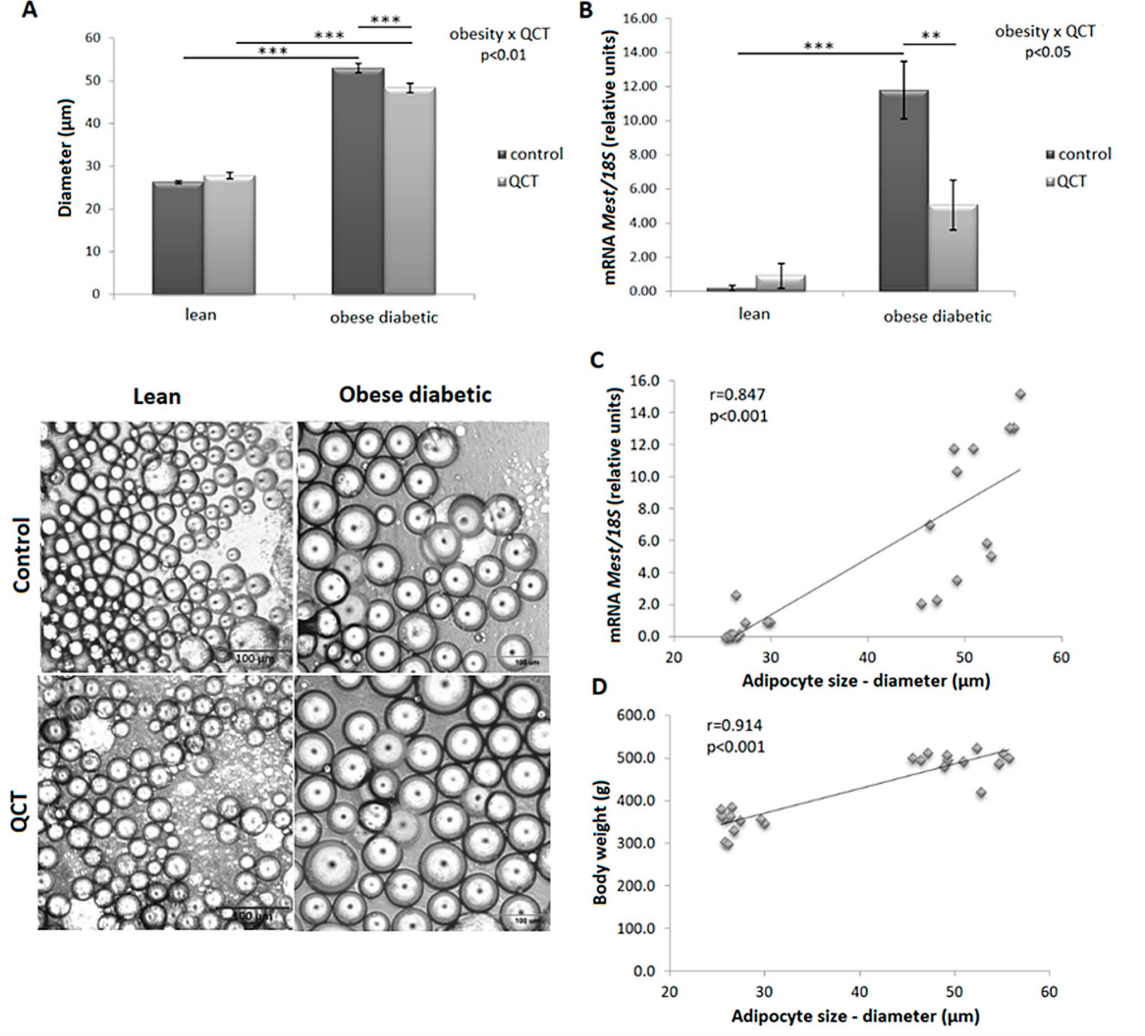
Effect of quercetin administration on the diameter of adipocytes obtained from subcutaneous adipose tissue in lean and obese ZDF rats (**A**). The gene expression of the adipocyte size marker *Mest* (mesoderm-specific transcription homologous protein) was normalized to 18 S mRNA expression (**B**). Results are presented as mean ± standard error of the mean. Data were analyzed using a two-way ANOVA with main factors obesity and QCT treatment. Correlation between *Mest* expression and adipocyte diameter (**C**), and between body weight and adipocyte diameter (**D**) was analyzed by Pearson’s correlation test. Level of statistical significance *p < 0.05; **p < 0.01; ***p < 0.001

The diameter of adipocytes obtained from ScWAT was significantly increased in obese ZDF rats compared to their lean controls in both treated and untreated groups. At the same time, in the group of obese ZDF rats, the diameter of adipocytes was significantly reduced due to administration of QCT (Fig. 1A). As expected, the size of the adipocytes correlated with the body weight of the experimental animals (Fig. 1D). The gene expression of the adipocyte size marker mesoderm-specific transcription homologous protein (*Mest*) was altered by phenotype as well as by QCT administration. Two-factor ANOVA revealed a significant interaction of these two factors (*p* < 0.05). In the group of control animals, we detected an increase in *Mest* expression due to obesity (*p* < 0.001), while in the group of animals treated with QCT, no significant changes due to obesity were observed. Administration of QCT decreased *Mest* expression in obese (*p* < 0.01) but not in lean ZDF rats (Fig. 1B). These changes were also consistent with the changes in adipocyte size, as *Mest* expression was positively correlated with adipocyte diameter (*r* = 0.847, *p* < 0.001) (Fig. 1C). In visceral rWAT, where fat cell size was not measured, we observed significantly increased *Mest* expression due to the obesity factor (*p* < 0.001), but changes after QCT treatment did not reach a statistically significant level (Table 3).

**Table 3:** The effect of quercetin (QCT) administration on the gene expression of retroperitoneal adipose tissue metabolism markers in lean and obese ZDF rats

mRNA	Lean		Obese diabetic		Main factor		
	Control *n* = 5	Quercetin *n* = 5	Control *n* = 6	Quercetin *n* = 6	Obesity	QCT	Inter-action
*aP2/18S* (r.u.)	2.52 ± 0.44	1.82 ± 0.14	2.93 ± 0.22	2.67 ± 0.22	*p* < 0.05	n.s.	n.s.
*Pparγ/18S* (r.u.)	2.80 ± 0.53	2.02 ± 0.29	3.18 ± 0.25	2.42 ± 0.18	n.s.	*p* < 0.05	n.s.
*Mest/18S* (r.u.)	0.82 ± 0.234	0.66 ± 0.11	0.31 ± 0.05	0.16 ± 0.04	*p* < 0.001	n.s.	n.s.
*Lpl/18S* (r.u.)	3.04 ± 0.55	2.13 ± 0.19	3.76 ± 0.44	3.32 ± 0.38	*p* < 0.05	n.s.	n.s.
*Plin/18S* (r.u.)	2.88 ± 0.36	2.50 ± 0.16	2.88 ± 0.19	2.83 ± 0.21	n.s.	n.s.	n.s.
*Fas/18S* (r.u.)	3.70 ± 1.18	1.93 ± 0.77	2.17 ± 0.27	3.21 ± 0.93	n.s.	n.s.	n.s.
*Hsl/18S* (r.u.)	2.91 ± 0.40	2.51 ± 0.31	2.84 ± 0.16	2.89 ± 0.36	n.s.	n.s.	n.s.
*F4/80/18S* (r.u.)	1.36 ± 0.21	2.70 ± 0.43	5.80 ± 0.49 ***	4.68 ± 0.77 *	*p* < 0.001	n.s.	*p* < 0.05
*Tnfα/18S* (r.u.)	2.16 ± 0.39	1.93 ± 0.29	4.02 ± 0.38	2.89 ± 0.33	*p* < 0.001	*p* < 0.05	n.s.
*p22/18S* (r.u.)	0.75 ± 0.20	0.78 ± 0.09	2.84 ± 0.16	2.89 ± 0.36	*p* < 0.001	n.s.	n.s.
*Nox2/18S* (r.u.)	0.72 ± 0.13	0.58 ± 0.03	3.95 ± 0.62	2.92 ± 0.42	*p* < 0.001	n.s.	n.s.
*Nox4/18S* (r.u.)	1.58 ± 0.57	1.56 ± 0.24	2.69 ± 0.13	2.65 ± 0.28	*p* < 0.01	n.s.	n.s.
*Adipoq/18S* (r.u.)	4.23 ± 0.82	3.31 ± 0.27	3.26 ± 0.18	3.34 ± 0.32	n.s.	n.s.	n.s.
*Pgc1α/18S* (r.u.)	0.55 ± 0.09	0.77 ± 0.05	0.61 ± 0.10	0.73 ± 0.21	n.s.	n.s.	n.s.

The expression of measured genes was normalized to 18 S mRNA expression (relative units, r.u.) and results are presented as mean ± SEM. Data were analyzed using a two-factor ANOVA with main factors obesity and QCT treatment. *Influence of obesity on the given parameter within the same experimental group (control or QCT), *p < 0.05; ***p < 0.001

**Fig. 2:**
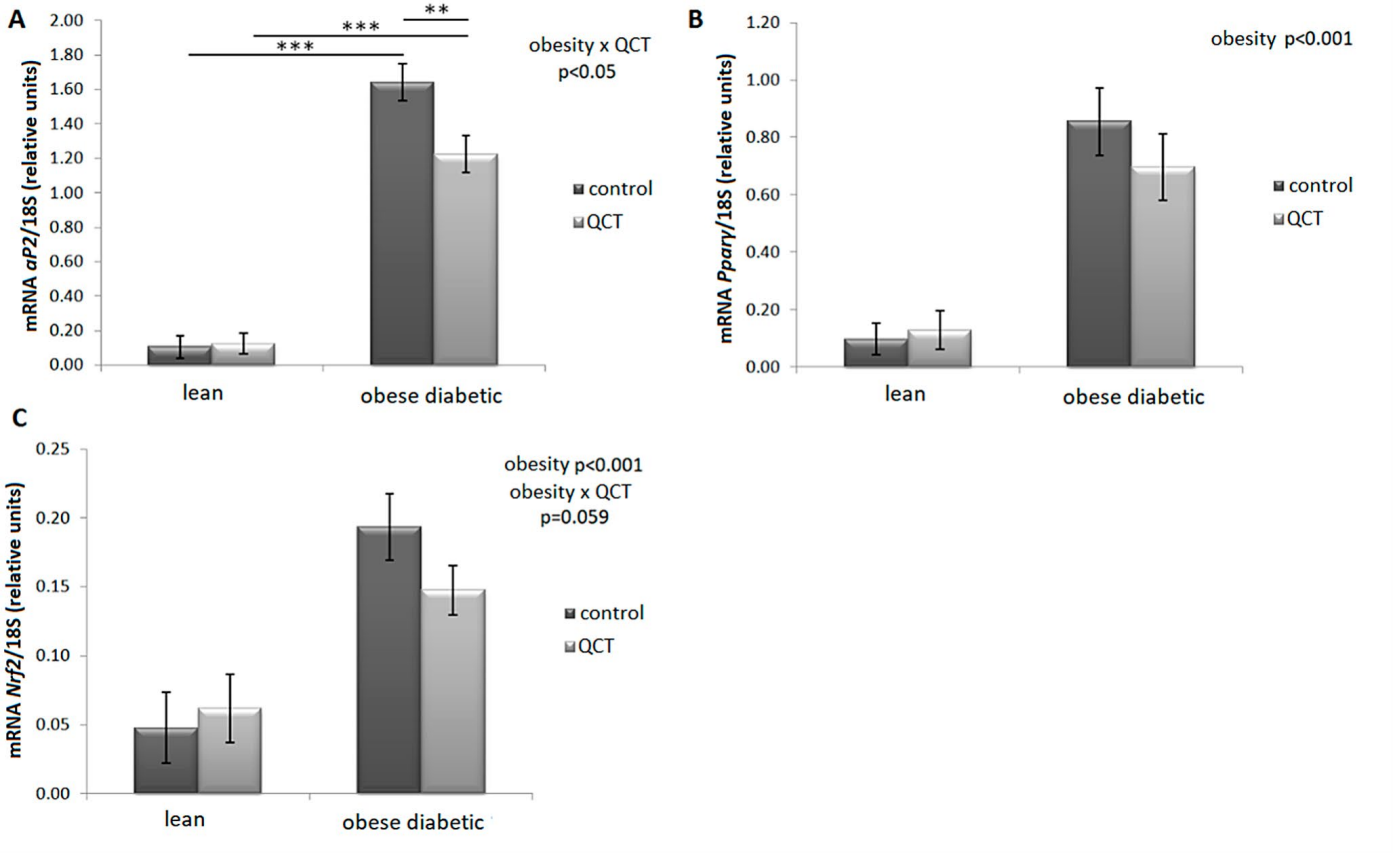
Effect of quercetin administration on the gene expression of adipocyte differentiation markers in subcutaneous adipose tissue in lean and obese ZDF rats. Expression of fatty acid-binding protein (*aP2*) (**A**), peroxisome proliferator-activated receptor γ (*Pparγ*) (**B**), and nuclear factor-related factor 2 (*Nrf2*) (**C**) was normalized to 18 S mRNA expression. Results are presented as mean ± standard error of the mean. Data were analyzed using a two-way ANOVA with main factors obesity and QCT treatment. Level of statistical significance *p < 0.05; **p < 0.01; ***p < 0.001

The gene expression of *aP2* was significantly altered by the interaction of two main factors - obesity and QCT (*p* < 0.05) in ScWAT. Due to the obese phenotype, there was a significant increase in the expression of *aP2* in rats treated (*p* < 0.001) and not treated with QCT (*p* < 0.001) (Fig. 2A). In the group of obese ZDF rats, administration of QCT significantly reduced *aP2* expression (*p* < 0.01). Gene expressions of *Pparγ* and *Nrf2* were increased by obesity in both experimental groups (*p* < 0.001) (Fig. 2B, C). However, in the case of obese ZDF rats a tendency (*p* = 0.059) to decreased expression of *Nrf2* was noticed after treatment with QCT.

**Fig. 3:**
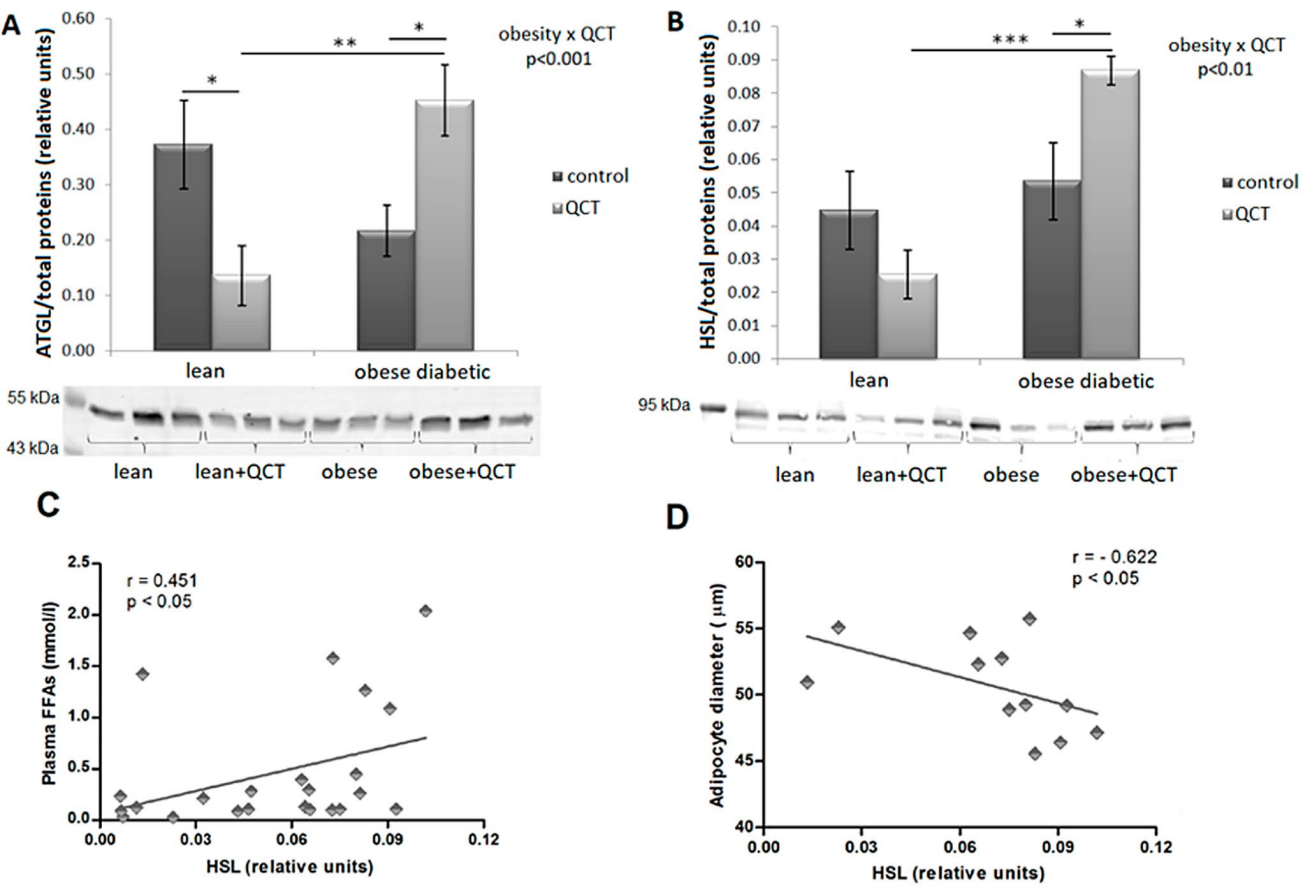
The effect of quercetin administration on the protein expression of lipolytic enzymes in subcutaneous adipose tissue in lean and obese ZDF rats. The expression of adipose triglyceride lipase (ATGL) (**A**) and hormone sensitive lipase (HSL) (**B**) was determined by western blot analysis. Data were normalized to total stained proteins. Results, presented as mean ± standard error of the mean, were analyzed using a two-way ANOVA with main factors obesity and QCT treatment. Correlation between HSL expression, plasma free fatty acids and adipocyte diameter was analyzed by Spearman’s correlation test (**C**, **D**). Level of statistical significance *p < 0.05; **p < 0.01; ***p < 0.001

Statistical analysis revealed a significant interaction of two factors - obesity and administration of QCT on the protein expression of ATGL in ScWAT of ZDF rats (*p* < 0.001) (Fig. 3A). In lean animals, QCT reduces ATGL protein level compared to the control group (*p* < 0.05), while in obese animals, administration of QCT induces the opposite effect on the ATGL expression (*p* < 0.05). HSL expression undergoes similar changes (Fig. 3B). A significant interaction of the two main factors (*p* < 0.01) was detected. In the group of animals receiving QCT, the HSL content was significantly increased due to obesity (*p* < 0.001). The protein expression of HSL was significantly increased by QCT supplementation in the group of obese animals (*p* < 0.05). Moreover, HSL expression was positively correlated with plasma levels of FFAs (*r* = 0.451; *p* < 0.05) (Fig. 3C) and negatively correlated with adipocyte size in ScWAT in obese animals (*r*=-0.622; *p* < 0.05) (Fig. 3D).

**Fig. 4:**
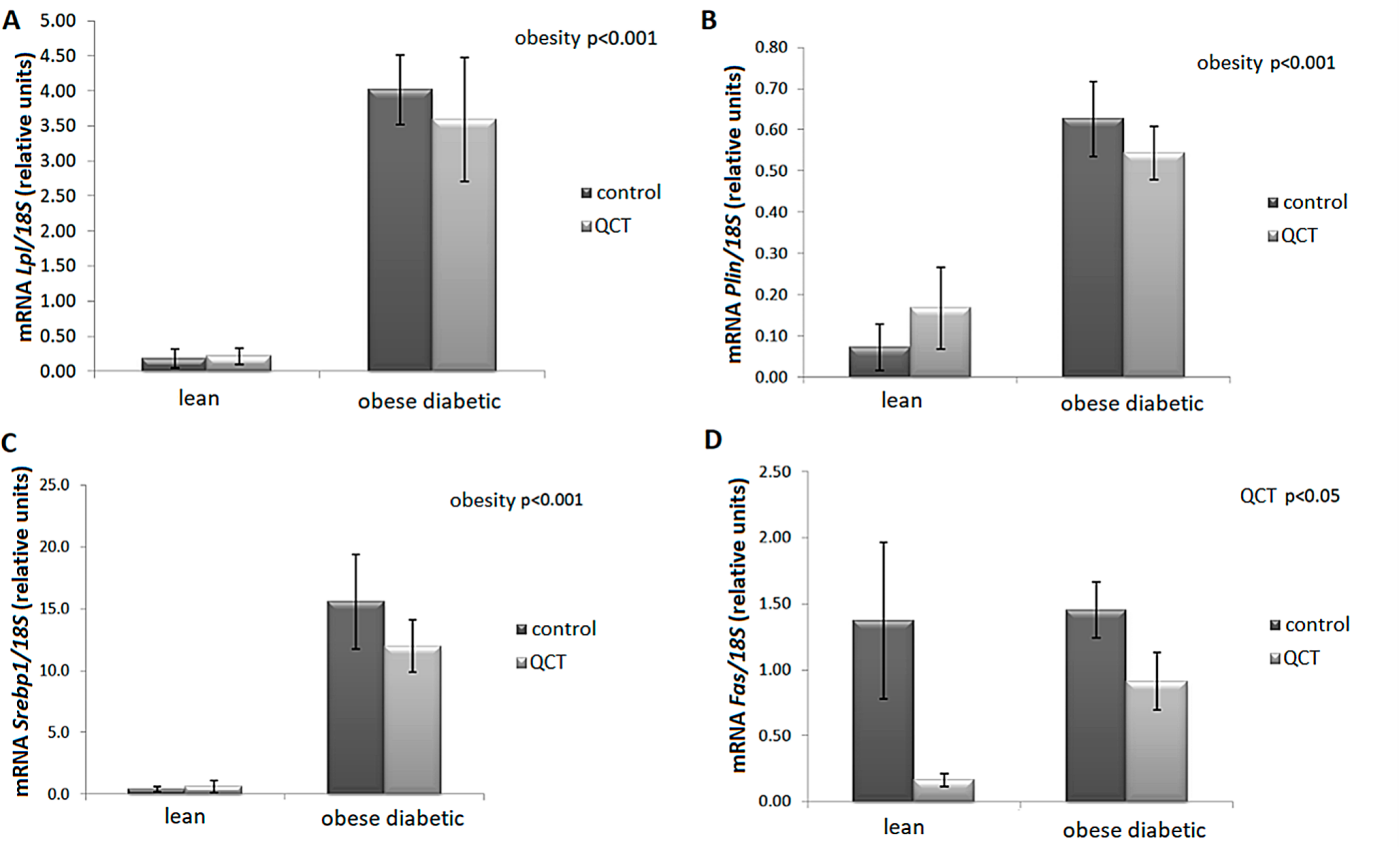
Effect of quercetin administration on the gene expression of lipogenic genes in subcutaneous adipose tissue in lean and obese ZDF rats. Expression of lipoprotein lipase (*Lpl*) (**A**), perilipin (*Plin*) (**B**), sterol regulatory element binding protein (*Srebp1*) (**C**) and fatty acid synthase (*Fas*) (**D**) was normalized to 18 S mRNA expression. Results are presented as mean ± standard error of the mean. Data were analyzed using a two-way ANOVA with main factors obesity and QCT treatment. Level of statistical significance *p < 0.05; **p < 0.01; ***p < 0.001

The gene expressions of *Lpl*,* Plin* and *Srebp1*, but not *Fas*, were significantly increased due to the obese phenotype, while administration of QCT had no significant effect on the observed gene expressions (Fig. 4A, B,C). However, QCT significantly reduced *Fas* mRNA levels (*p* < 0.05) in ScWAT in both lean and obese ZDF rats (Fig. 4D). In rWAT, *Lpl* and *aP2* gene expressions were significantly increased by obesity (*p* < 0.05), without significant influence of QCT administration. Neither factor obesity nor factor QCT treatment had an effect on gene expression of *Plin*, *Fas*, and *Hsl* in visceral fat tissue. We further found an inhibitory effect of QCT treatment on *PPARγ* gene expression in rWAT (*p* < 0.05) ((Table 3).

**Fig. 5:**
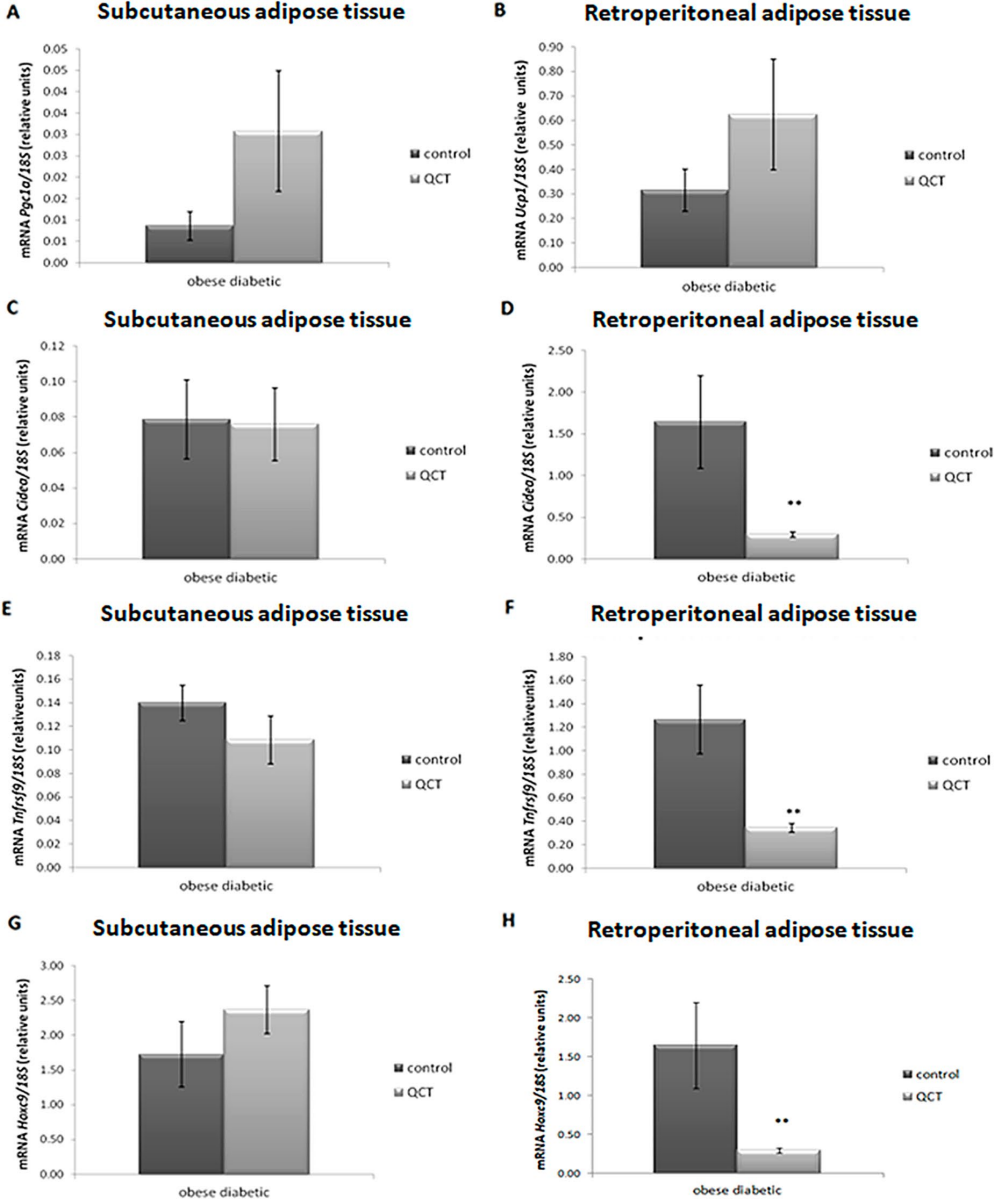
Effect of quercetin administration on the gene expression of brown, beige and white adipocyte markers in subcutaneous and retroperitoneal adipose tissue in obese ZDF rats. Expression of peroxisome proliferator-activated receptor coactivator 1α (*Pgc1α*) (**A**), uncoupling protein 1 (*Ucp1*) (**B**), cell death-inducing DNA fragmentation factor alpha-like effector A (*Cidea*) (**C**,** D**), a member of the tumour necrosis factor receptor superfamily member 9 (*Tnfrsf9*) (**E**,** F**) and homeobox C9 (*Hoxc9*) (**G**,** H**) was normalized to 18 S mRNA expression. Results are presented as mean ± standard error of the mean. Data were analyzed using a one-way ANOVA and level of statistical significance **p < 0.01

To determine the effect of QCT administration on adipocyte transdifferentiation, markers of brown (*Ucp1*,* Pgc1α*,* Cidea*), beige (*Ucp1*,* Pgc1α*,* Tnfrsf9*) and white (*Hoxc9*) adipocytes were determined in ScWAT and also rWAT depot. The gene expression of these markers, except *Hoxc9*, was detectable only in obese ZDF rats, while in lean ZDF rats it was below the limit of detection. We noted an increased expression of the mitochondrial biogenesis marker - peroxisome proliferator-activated receptor 1α coactivator (*Pgc1α*) by QCT administration at the limit of significance (*p* = 0.08) only in ScWAT (Fig. 5A). The QCT application had no significant effect on uncoupling protein 1 (*Ucp1*) expression in rWAT (Fig. 5B). Gene expression of markers of brown (cell death-inducing DNA fragmentation factor alpha-like effector A, *Cidea*), beige (a member of the tumour necrosis factor receptor superfamily member 9, *Tnfrsf9*) and white (homeobox C9, *Hoxc9*) adipocytes was without significant influence of QCT in ScWAT in the group of obese ZDF rats (Fig. 5C, E and G). In contrast, in rWAT the expression of all three markers was significantly decreased by administration of QCT (*Cideap* < 0.01, *Tnfrsf9p* < 0.01 and *Hoxc9p* < 0.01) compared to untreated obese ZDF rats (Fig. 5D, F and H).

**Fig. 6:**
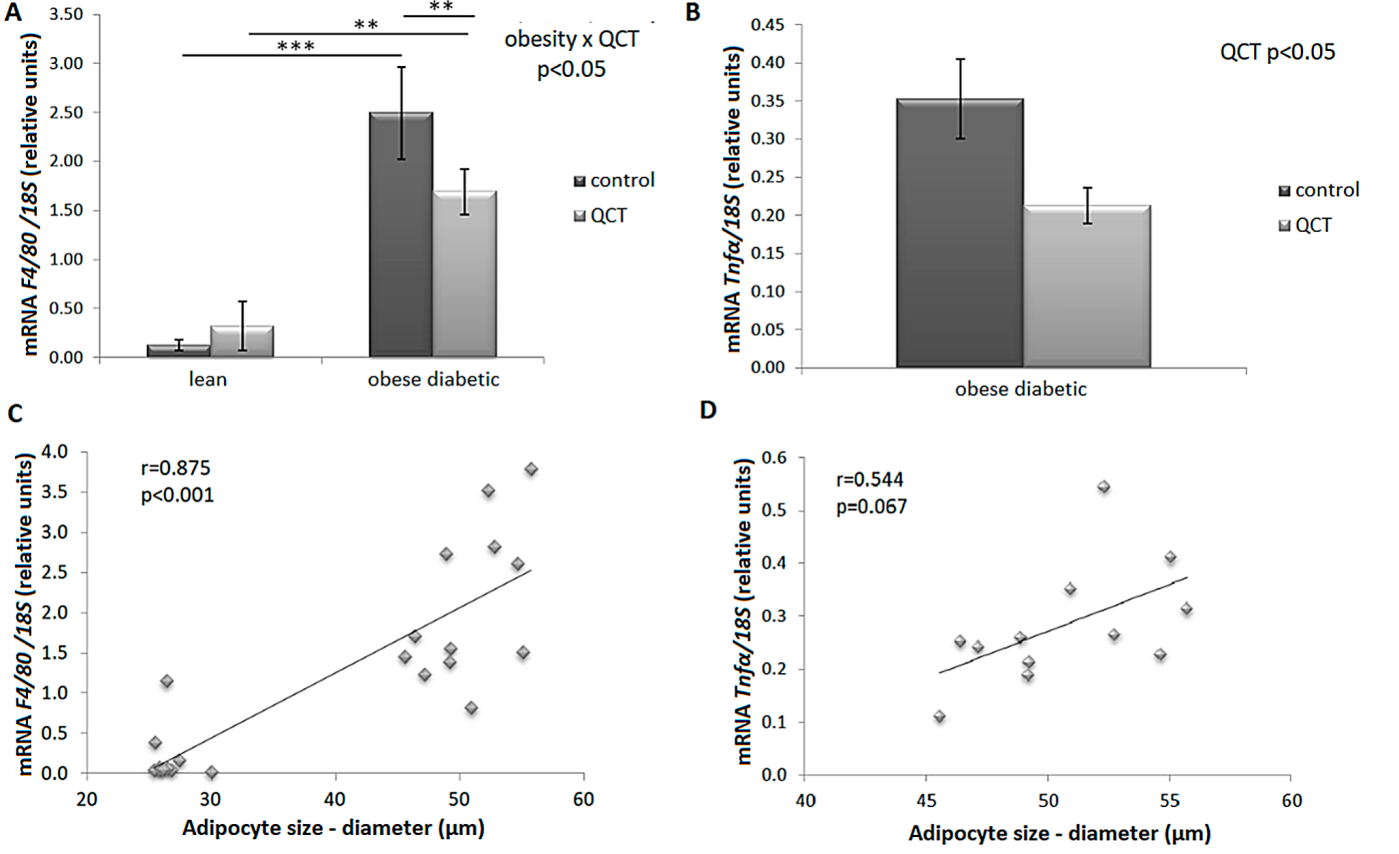
Effect of quercetin administration on the gene expression of inflammatory markers in subcutaneous adipose tissue in lean and obese ZDF rats. The expression of the macrophage marker - Adhesion G Protein-Coupled Receptor E1 (*F4/80*; *Adgre1*) (**A**) and tumour necrosis factor (*Tnfα*) (**B**) was normalized to the expression of 18 S mRNA. Results are presented as mean ± standard error of the mean. Data were analyzed using a two-way ANOVA with main factors obesity and QCT treatment. Correlation between *F4/80*, *Tnfα* expression and adipocyte diameter was analyzed by Pearson’s correlation test (**C**, **D**). Level of statistical significance *p < 0.05; **p < 0.01; ***p < 0.001

Using a two-way analysis of variance, a significant interaction (*p* < 0.05) of the factors obesity and QCT administration on the gene expression of the marker of macrophage infiltration into adipose tissue was detected (Fig. 6A). In ScWAT, a significant increase in *F4/80* mRNA levels was observed in the control (*p* < 0.001) as well as in QCT treated group (*p* < 0.01) due to obese phenotype. In the group of obese ZDF rats, administration of QCT significantly decreased the expression of *F4/80* (*p* < 0.01). The *Tnfα* mRNA levels were undetectable in the group of lean animals. In obese diabetic rats, a significant decrease in *Tnfα* expression was noted due to QCT administration (*p* < 0.05) (Fig. 6B). These declines in *F4/80* and *Tnfα* expressions in QCT-treated obese rats positively correlated with the size of adipocytes in ScWAT (Fig. 6C, D). In visceral rWAT, the gene expression of *F4/80* was altered by obesity but not by QCT treatment and two-factor ANOVA revealed a significant interaction of these factors (*p* < 0.05). We detected significant increase in *F4/80* expression due to obesity (*p* < 0.001) in the group of control animals as well as in the group of QCT-treated rats (Table 3). We noticed a significant increase in *Tnfα* gene expression due to obese phenotype in rWAT in ZDF rats (*p* < 0.001) and significant decrease in *Tnfα* expression by the influence of QCT administration (*p* < 0.05).

**Fig. 7:**
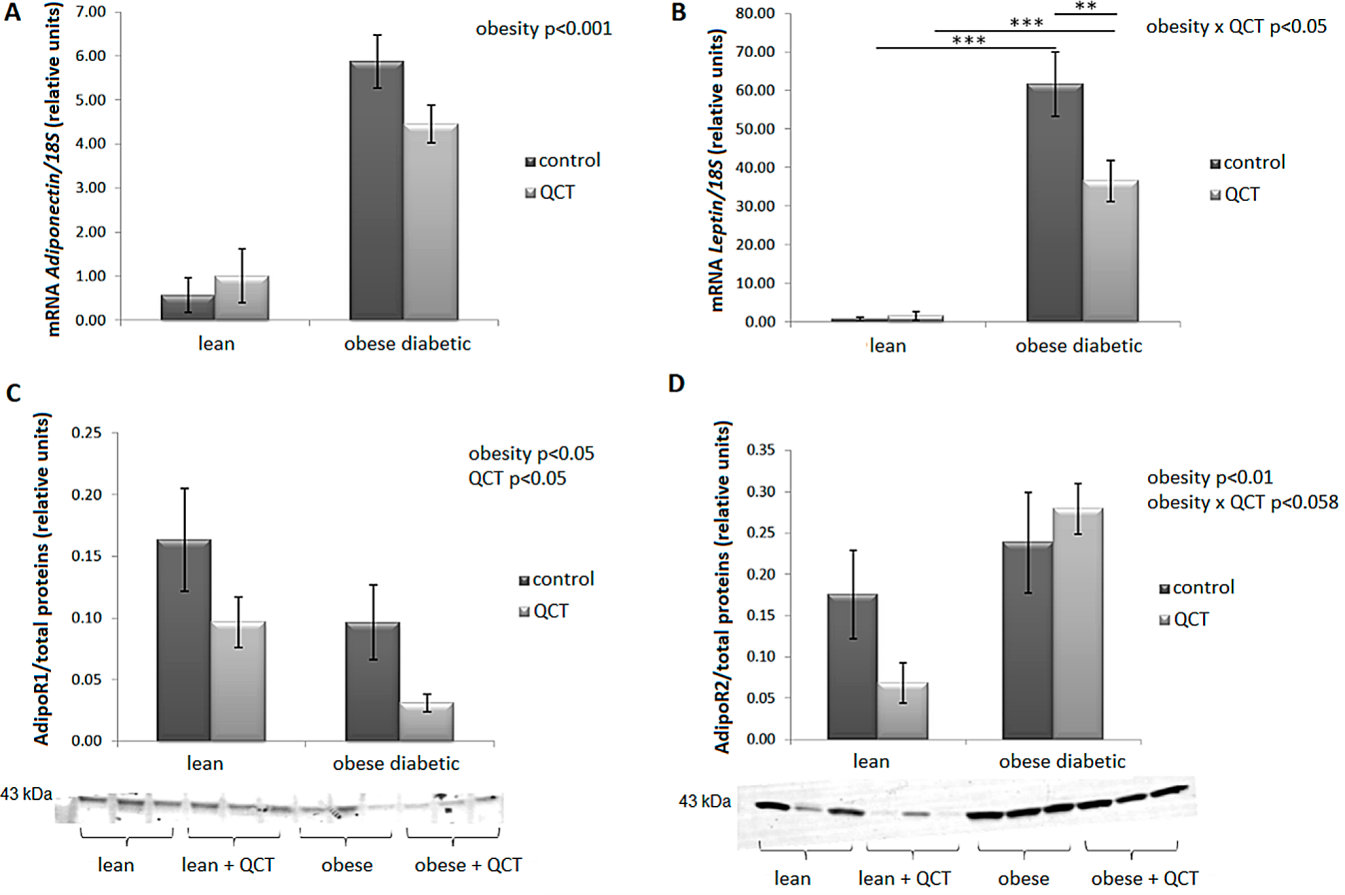
The effect of quercetin administration on the expression of adipokines in subcutaneous adipose tissue in lean and obese ZDF rats. The gene expression of adiponectin (*Adipoq*) (**A**) and leptin (*Lep*) (**B**) were normalized with respect to 18 S mRNA expression. Protein expression of adiponectin receptor 1 (AdipoR1) (**C**) and AdipoR2 (**D**) was determined using western blot analysis. Data were normalized to total stained proteins. Results are presented as mean ± standard error of the mean. Data were analyzed using a two-way ANOVA with main factors obesity and QCT treatment. Level of statistical significance *p < 0.05; **p < 0.01; ***p < 0.001

Adiponectin (*Adipoq*) gene expression was significantly increased by obesity (*p* < 0.001) in ScWAT, without significant influence of QCT administration (Fig. 7A). In the case of leptin (*Lep*), a significant interaction of two main factors (*p* < 0.05) was detected. Due to the effect of obesity, *Lep* mRNA levels increased in both groups (*p* < 0.001), while in the group of obese diabetic rats, the increase was significantly lower due to the effect of QCT administration (*p* < 0.01) (Fig. 7B). Protein content of adiponectin receptor 1 (AdipoR1) in ScWAT was significantly reduced by obesity (*p* < 0.05) as well as by QCT administration (*p* < 0.05) (Fig. 7C). Conversely, AdipoR2 protein content increased with obesity (*p* < 0.01). Moreover, we noted a borderline significance of the interaction of the two main factors (*p* = 0.058), whereby in the group of lean animals, QCT tended to reduce the expression of AdipoR2, which did not occur in the group of obese ZDF rats (Fig. 7D).

**Fig. 8:**
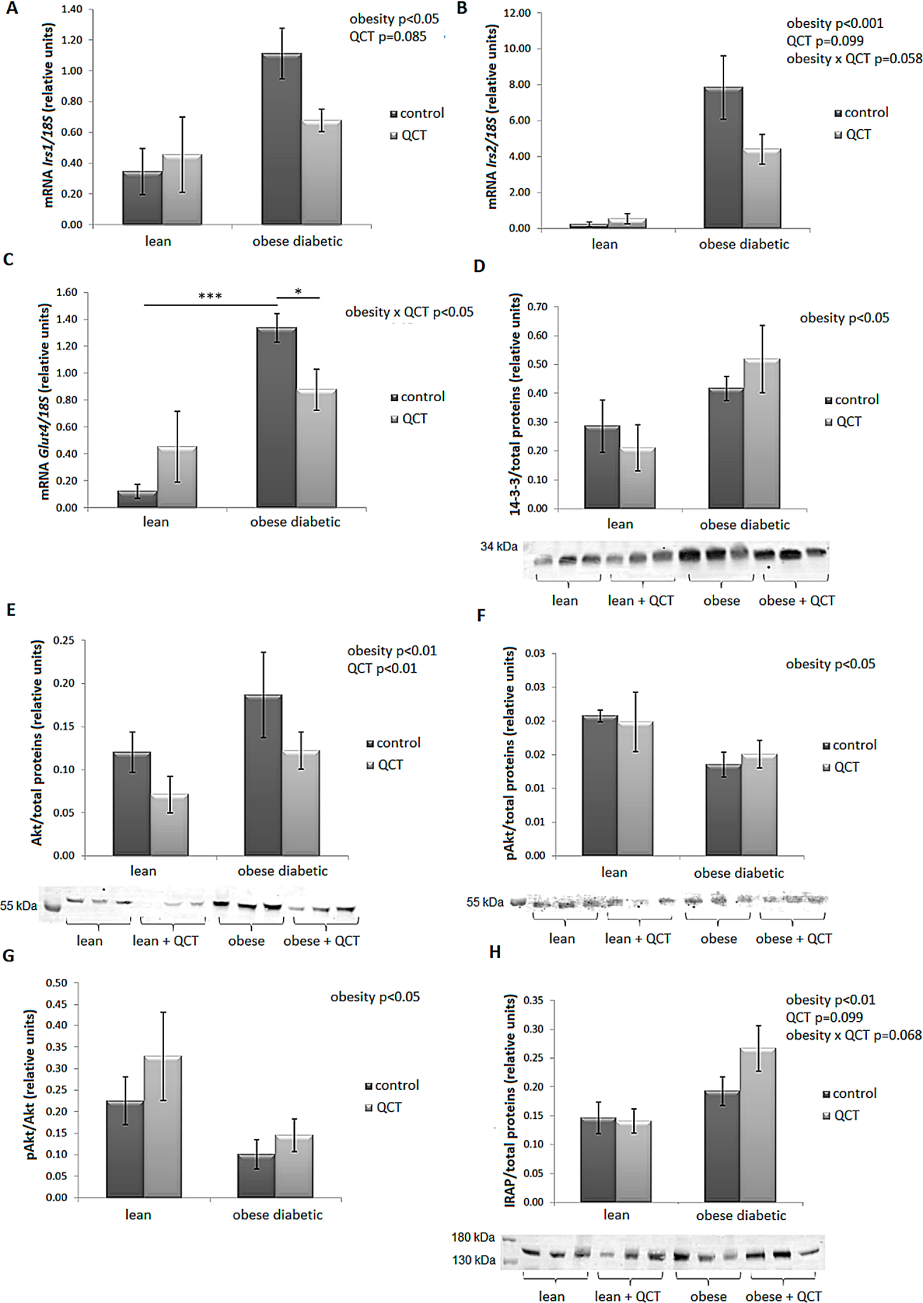
Effect of quercetin administration on the expression of genes and proteins involved in the insulin signaling cascade in subcutaneous adipose tissue in lean and obese ZDF rats. The gene xpression of insulin receptor substrate 1 (*Irs1*) (**A**), *Irs2*(**B**) and glucose transporter 4 (*Glut4*) (**C**) was normalized to 18 S mRNA expression. Protein expression of 14-3-3 (**D**), Akt kinase (**E**), phosphorylated Akt kinase at threonine residue Thr308 (pAkt) (**F**) and insulin-regulated aminopeptidase (IRAP) (**H**) was determined by western blot analysis. Figure G shows the ratio of pAkt and total Akt (**G**). Data were normalized to total stained proteins. Results are presented as mean ± standard error of the mean. Data were analyzed using a two-way ANOVA with main factors obesity and QCT treatment. Level of statistical significance *p < 0.05; **p < 0.01; ***p < 0.001

The gene expression of *Irs1* was significantly increased by obesity (*p* < 0.05). The expression of *Irs1* tended to decrease due to administration of QCT (*p* = 0.085) (Fig. 8A). Similarly, a significant increase in *Irs2* mRNA levels was detected by the obese diabetic phenotype (*p* < 0.05) (Fig. 8B). Administration of QCT showed a trend to decrease *Irs2* expression (*p* = 0.099), and a borderline significance of the interaction of the two main factors (*p* = 0.058) was detected. A significant interaction between the factors obesity and QCT therapy was demonstrated in *Glut4* expression (*p* < 0.05) (Fig. 8C). In the control animals, there was a significant increase in *Glut4* mRNA levels due to obesity (*p* < 0.001), which did not occur in animals treated with QCT. Therefore, *Glut4* expression was significantly lower after QCT therapy in the group of obese ZDF rats compared to their controls with the same phenotype (*p* < 0.05). The 14-3-3 protein content in ScWAT was significantly increased by obesity, without significant influence by QCT administration (Fig. 8D). Protein expression of Akt kinase was significantly increased by obesity (*p* < 0.01), while administration of QCT had the opposite effect, i.e., there was a significant decrease in Akt protein content in both lean and obese ZDF rats (*p* < 0.05) (Fig. 8E). Akt kinase phosphorylation on the threonine residue (*p* < 0.05) (Fig. 8F) as well as the ratio of phosphorylated to total Akt kinase were significantly reduced by obesity (*p* < 0.05) (Fig. 8G). The expression of insulin-regulated aminopeptidase (IRAP) at the protein level was significantly increased by the obese phenotype (*p* < 0.01) and showed a tendency to further increase after QCT administration in the ScWAT of obese ZDF rats (*p* = 0.068) (Fig. 8H).

**Fig. 9:**
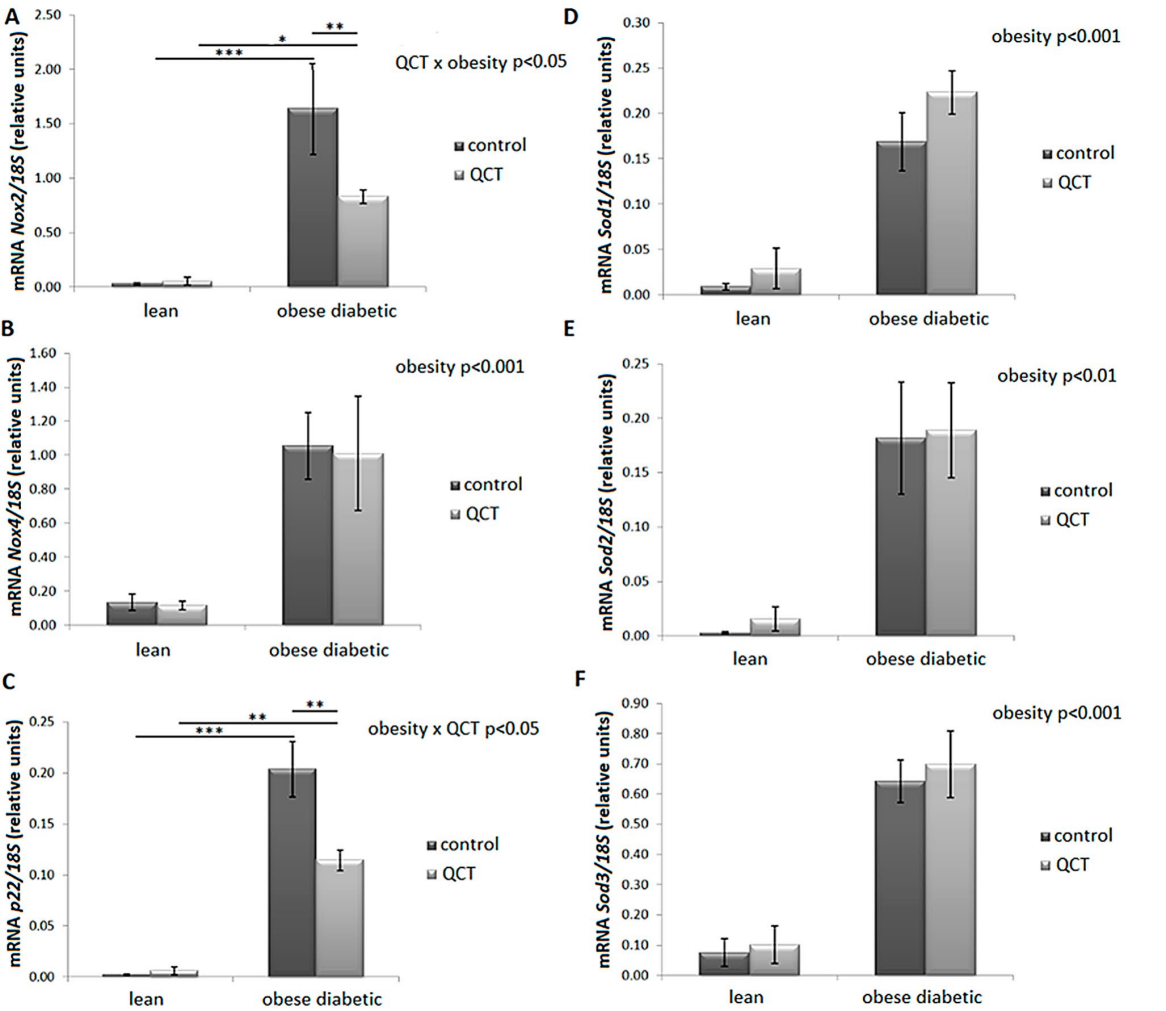
Effect of quercetin administration on the gene expression of oxidative stress markers and antioxidant enzymes in subcutaneous adipose tissue in lean and obese ZDF rats. Expression of reduced nicotinamide adenine dinucleotide phosphate oxidase 2 (*Nox2*) (**A**), *Nox4*(**B**), *p22*(**C**), superoxide dismutase 1 (*Sod1*) (**D**), *Sod2*(**E**) and *Sod3*(**F**) was normalized to 18 S mRNA expression. Results are presented as mean ± standard error of the mean. Data were analyzed using a two-way ANOVA with main factors obesity and QCT treatment. Level of statistical significance *p < 0.05; **p < 0.01; ***p < 0.001

The gene expression of the pro-oxidant gene *Nox4* (*p* < 0.001) was significantly increased by obesity in ScWAT (Fig. 9B). Regarding *Nox2* mRNA level, the interaction of two main factors - obesity and QCT therapy (*p* < 0.05) was detected, where administration of QCT decreased *Nox2* expression in the group of obese rats, but not in lean animals (Fig. 9A). The *p22* mRNA level was significantly increased by the obese phenotype in both control (*p* < 0.01) and QCT-treated animals (*p* < 0.001). Administration of QCT significantly decreased the expression of *p22* in the group of obese diabetic ZDF rats (*p* < 0.01), but not in lean animals (Fig. 9C). A significant effect of obesity was detected on gene expression of antioxidant enzymes superoxide dismutases 1, 2 and 3 (*Sod1*, *Sod2* and *Sod3*), since there was a significant increase in their mRNA levels in ScWAT in obese animals. Administration of QCT did not affect the expression of these genes (Fig. 9D, E,F). In rWAT, two-way ANOVA revealed statistically significant increase in *p22* (*p* < 0.001), *Nox2* (*p* < 0.001) and *Nox4* (*p* < 0.01) expression due to obesity but administration of QCT did not significantly affect the expression of these genes (Table 3).

**Fig. 10:**
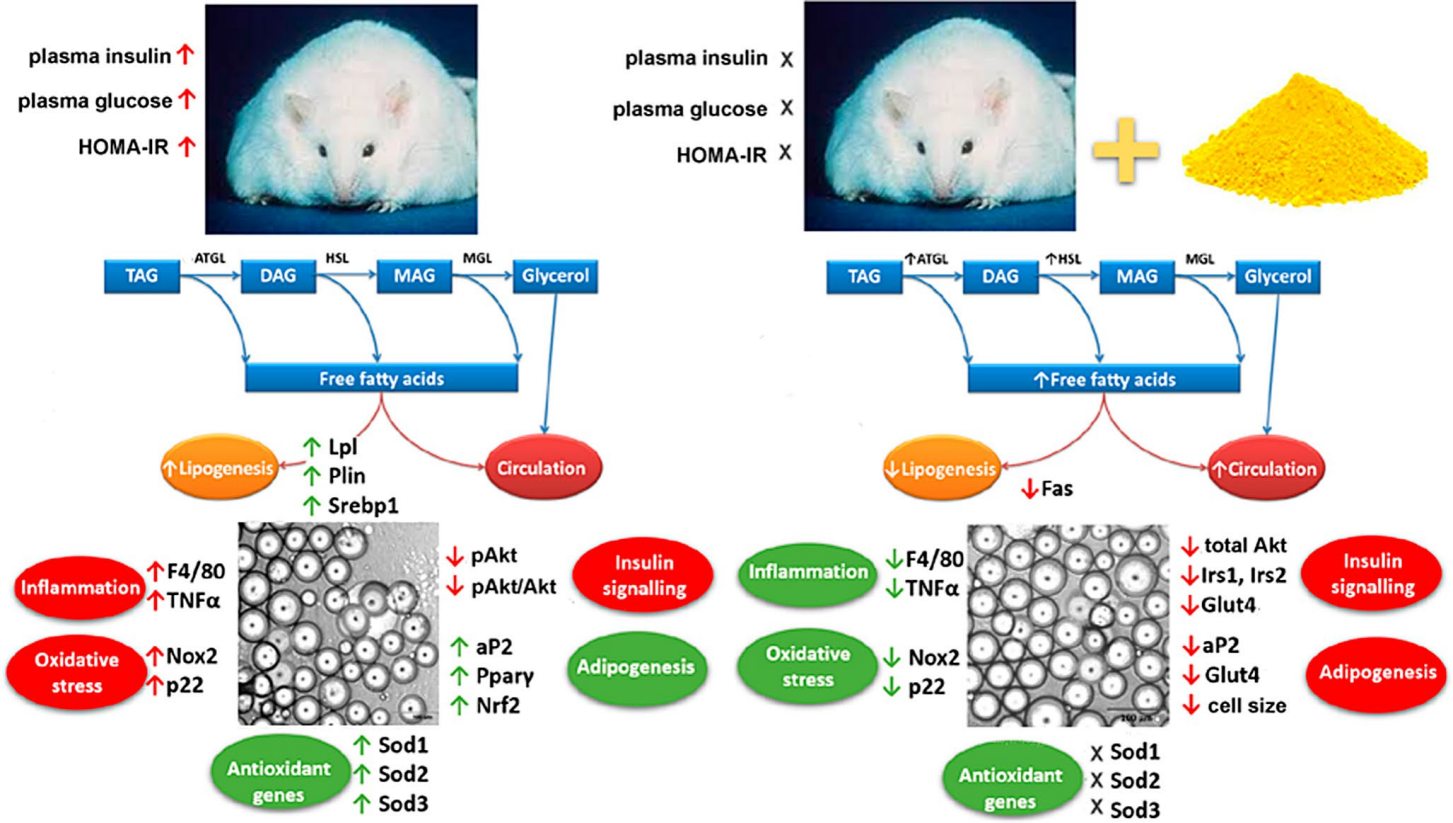
Schematic overview of the investigated parameters in subcutaneous adipose tissue due to obesity and diabetes (left, compared to lean cotrol) and quercetin administration (right, compared to untreated obese rats). Gene expressions were made by PCR and protein expression by Western blotting. In obesity, enlargement of adipose tissue is accompanied by stimulated lipogenesis and adipogenesis in order to store the excess supply of nutrients in fat stores. The plasmatic levels of free fatty acids (FFAs) are not affected. As a result, there is an increase in the size of adipocytes, which is closely associated with the development of inflammation by increasing the expression of the macrophage infiltration marker (*F4/80*) and the proinflammatory cytokine tumour necrosis factor (*Tnfα*). Interestingly, not only the expression of pro-oxidant genes (reduced nicotinamide adenine dinucleotide phosphate oxidase 2 and 4, *Nox2* and, *Nox4*; subunits of the NADPH oxidase complex, *p22*), but also anti-oxidant genes (superoxide dismutases, *Sod1, Sod2, Sod3*) is up-regulated. Due to obesity and diabetes, phosphorylation of one of the key proteins of the insulin signaling cascade (Akt kinase) is suppressed in subcutaneous adipose tissue. After administration of quercetin to obese ZDF rats, lipolysis is stimulated by increasing the protein expression of adipose triglyceride lipase (ATGL) and hormone sensitive lipase (HSL), and an increased release of FFAs is also reflected in the circulation. They do not undergo lipogenesis due to the inhibited expression of fatty acid synthase (*Fas*) and adipogenesis (fatty acid binding protein, *aP2*; glucose transporter, *Glut4*). These changes lead to a decrease in the size of adipocytes, which is however accompanied by a decrease in the expression of inflammatory markers and pro-oxidant genes. It suggests that the reduction in the adipocyte size is not conditioned by the stimulation of hyperplastic growth of adipose tissue. There is also worsening in the gene expression (insulin receptor substrate 1 and 2, *Irs1* and *Irs2*) and protein level (total Akt) of components of the insulin signaling cascade due to administration of quercetin, although Akt phosphorylation was not affected by treatment. The mRNA expressions would require measurement of protein and/or activity expressions for better description of physiological function in the further experiments. Peroxisome proliferator-activated receptor γ, *Ppar* γ; nuclear factor-related factor 2, *Nrf2*; ↓ - down-regulated by obesity or quercetin treatment; ↑ - up-regulated by obesity or quercetin treatment; X – not affected by quercetin

## Discussion

Through the production of bioactive adipokines, adipose tissue can affect the metabolism of the whole organism regulating food intake, glucose metabolism and insulin signaling [8, 24]. During the development of obesity and type 2 diabetes mellitus, the redox balance is disturbed and subchronic inflammation develops in adipose tissue [8, 25]. Several studies suggest beneficial effect of flavonoid administration on glucose homeostasis, reduction of adiposity and inflammation during the development of these metabolic disorders [26, 27]. In our study, we focused on the effect of QCT on adipogenesis, lipid and glucose metabolism, adipokines production, oxidative status and inflammation in white adipose tissue in vivo in obese diabetic ZDF rats.

We confirmed an altered metabolic profile in Zucker diabetic fatty rats compared to their lean controls but we did not reveal a significant effect of QCT administration on the observed parameters. ZDF rats had hyperinsulinemia, hyperglycemia, hyperleptinemia, increased HOMA-IR and adiposity index. Nevertheless, we did not observe increased plasma levels of FFAs in obese non-treated subjects. However, level of circulating FFAs increased up to 6-fold in obese ZDF rats after QCT administration. We have previously shoved that plasma triglycerides and cholesterol levels do not display differences between untreated obese ZDF rats and those who were administered QCT [14]. In order to verify the origin of the increased levels of FFAs, the size of adipocytes in the subcutaneous adipose tissue was determined. We found that the adipocyte size was significantly reduced in the group of obese rats due to QCT administration. The reduction in adipocyte size was also confirmed by the gene expression of *Mest*, which promotes adipocyte enlargement and is a predictive marker of adipose tissue expansion [28]. *Mest* gene expression has been found increased in white adipose tissue in obese and diabetic mice compared to control group [29]. This is consistent with our results, as we observed a significant increase in *Mest* expression due to obesity in both ScWAT and visceral rWAT. In ScWAT, the gene expression of *Mest* is positively correlated with the size of adipocytes.

The adipogenic markers in ScWAT were determined to reveal whether the reason for the reduced diameter of adipocytes is the formation of new adipocytes from precursor cells. However, this hypothesis was not confirmed, since the gene expression of the main transcriptional factor *Pparγ* was not altered and markers of late phase of adipogenesis (*aP2*, *Glut4*, *Fas*) were suppressed by QCT administrationin in obese ZDF rats. This suggests rather an inhibitory effect of QCT on adipogenesis, which is consistent with other in vitro studies [15, 16, 30, 31] and also with our previously published in vitro study investigating the effect of QCT addition into the cell medium of primary rat preadipocytes isolated from ScWAT of Wistar rats [18]. During obesity, hypertrophic expansion of already existing adipocytes coupled with no adequate vascular system development subsequently causes hypoxia and subchronic inflammation in adipose tissue [25]. The anti-adipogenic effect of QCT is often reported and considered as beneficial, but in this case, the alterations in the function of adipose tissue as such occur and subsequently lead to impaired storage capacity of excess metabolic substrates. Thus, the concentrations of excess metabolic substrates increase in the plasma leading to ectopic fat deposition and lipotoxicity in non-adipose tissues such as liver and skeletal muscle [32]. However, our findings of increased serum levels of FFAs after QCT treatment in obese ZDF rats are not consistent with the results of the study by Rivera et al. [33] who observed significant decrease in plasma FFAs in obese Zucker rats after 10 weeks of QCT treatment in dose 2 or 10 mg/kg/day. This discrepancy with our findings might be caused by several facts, namely the different dose and duration of QCT treatment, and also that the obese Zucker rats had dyslipidemia and were not diabetic [33], whereas in our study, the obese diabetic animals did not suffer from increased levels of FFAs.

Our results show that the decrease in the adipocyte size was not caused by adipogenesis induction therefore the mechanism of adipocyte size reduction in the obese ScWAT by QCT was further investigated. We found that QCT significantly increases the protein content of lipolytic enzymes ATGL and HSL in the ScWAT of obese ZDF rats. Simultaneously, the protein expression of HSL correlated with the diameter of adipocytes and plasma levels of FFAs. This indicates to an increased lipolysis in adipose tissue in obese ZDF rats treated with QCT causing FFAs to be cleaved from triacylglycerols and released into the plasma. In addition, this release of FFAs may be enhanced by reduced lipogenesis as we found significantly inhibited adipose tissue *Fas* gene expression in obese ZDF rats treated with QCT. A combination of the three mechanisms mentioned above, i.e. inhibition of adipogenesis, stimulation of lipolysis and inhibition of lipogenesis, exerts substantial influence on ScWAT remodelling in obese ZDF rats treated with QCT.

The hypertrophic enlargement of adipocytes during obesity is nearly affiliated with the development of oxidative stress and subchronic inflammation of adipose tissue [25]. We found that QCT has anti-inflammatory effects in obese rats by suppressing the expression of adipose tissue markers of macrophages infiltration *F4/80* in ScWAT and pro-inflammatory cytokine *Tnfα* in both ScWAT and rWAT. Further, the levels of *F4/80* mRNA positively correlate with the adipocyte diameter in ScWAT. The lowering effect of QCT on Tnfα production by WAT was observed also in other studies in experimental models of metabolic dysfunctions [33, 34].

Adipose tissue lipid metabolism and adipogenesis are controlled by the redox shift. The redox balance reaches a higher degree in during the adipogenic differentiation [7, 8], which is due to the increased ROS production and also the activity of reducing agents. Also, with increased nutrient overload, both arms of the redox balance are shifted to a new level in response to the new metabolic demands caused by increased lipid storage. This is consistent with our results, as the expressions of the markers of oxidative stress and also antioxidative markers were profoundly increased by obesity. Furthermore, we have revealed the inhibitory effect of QCT administration on *Nox2* and *p22* expression in the group of obese animals. These results obtained from in vivo study showing suppressed expression of oxidative stress and also adipogenic markers after QCT treatment are consistent with our previously published in vitro results on differentiating primary rat preadipocytes, where QCT application reduced ROS generation in a dose-dependent manner and suppressed differentiation [18]. ROS originating from Nox4 play a key role in adipogenesis and it has been shown that NOX4-derived ROS play a role in the onset of insulin resistance [7, 35]. Interestingly, unlike *Nox2* and *p22*, we did not find *Nox4* expression downregulated after QCT administration in obese ZDF. Taken together, QCT administration disrupted the balance between the expression of pro-oxidant and oxidative genes. We suggest that disruption of this balance may have attenuated ROS production contributing to inhibition of adipogenesis and lipogenesis in ScWAT and subsequent release of FFAs into the circulation, a condition that causes ectopic lipid storage and impairs peripheral insulin sensitivity.

Obese ZDF rats suffer from both systemic and tissue insulin resistance. In order to determine the effect of QCT on the insulin sensitivity, HOMA-IR was calculated and the mRNA and/or protein level of components of insuling signaling cascade in ScWAT was measured. Since HOMA-IR and blood glucose level increased by obesity and diabetes was not changed by QCT administration, we conclude that the QCT in our experiment did not have a major effect on systemic insulin sensitivity. At the level of adipose tissue, phosphorylation of Akt kinase was suppressed by obesity, with no significant effect of QCT administration. The protein expressions of IRAP, 14-3-3 and total Akt were significantly increased by obesity in ZDF rats. These proteins play a key role in insulin signal transduction, basal retention of GLUT4-containing vesicles (GSVs) and insulin-stimulated mobilization of GSVs, which subsequently fuse with the plasma membrane, facilitating glucose uptake by adipocytes. Impairment of GSVs formation or retention may contribute to increased degradation of GLUT4 in lysosomes and shortening their half-life [36, 37]. We assume that the increased IRAP, 14-3-3 and total Akt proteins content together with increased *Irs1*, *Irs2* and *Glut4* gene transcription in obese ZDF rats indicates the activation of a compensatory mechanism to mitigate metabolic dysfunction in order to intensify the insulin signal transmission. Moreover, after QCT treatment the inhibitory effect on the gene expression of *Irs1* and *Glut4* and protein level of total Akt kinase was noted in obese ZDF rats. These changes may contribute to the aggravation of the insulin sensitivity of ScWAT in obese diabetic rats.

The ZDF rats, as a genetic model of obesity, exhibit hyperleptinemia and associated leptin resistance, which results in a deterioration in insulin sensitivity [38]. We investigated the effect of QCT administration on the plasma concentrations and ScWAT gene expressions of two dominant adipokines, leptin and adiponectin. We confirmed a significantly higher expression of *Lep* gene in obesity, which is also reflected in increased plasma levels of leptin. Partial suppression of *Lep* expression in subcutaneous depot after QCT treatment had no effect on circulating leptin levels and we consider that it is related to the reduction of adipocyte size. Neither obesity nor QCT treatment had an effect on the plasma levels of adiponectin. In contrast, at the mRNA level, we noted a significant increase of *Adipoq* expression in adipose tissue. This discrepancy can be explained either by increased degradation of adiponectin in obesity, or by post-transcriptional/post-translational modifications that have not been elucidated yet. The half-life of adiponectin is very short, and it can also act in an auto- or paracrine manner [39–41]. For this reason, we determined the presence of adiponectin receptors AdipoR1 and AdipoR2 protein in ScWAT. The adiponectin receptors, which mediate the insulin-sensitizing and antidiabetic actions of adiponectin, in adipose tissue stimulate lipid and glucose metabolism and have pro-/anti-inflammatory effects [41]. Knowledge about the AdipoR1/2 in adipose tissue in obesity and diabetes is scarce and not unambiguous. In insulin-resistant ob/ob mice, the expression levels of both receptors were substantially lowered in muscle and adipose tissue [42], whereas in human study, mRNA expression of *ADIPOR1*, but not *ADIPOR2* in skeletal muscle reflected first-phase of insulin secretion independently of insulin sensitivity and body fat [43]. In our study using ZDF rats as model of obesity and diabetes type II, AdipoR1 protein expression was significantly suppressed by metabolic disorders, which was further accentuated by the administration of QCT. On the contrary, AdipoR2 protein expression was higher in obese rats and unaffected by QCT treatment. Assuming a paracrine effect of adiponectin, the up-regulated adiponectin/AdipoR2 system in ScWAT in obese rats may represent a compensatory mechanism against insulin resistance and down-regulated AdipoR1 by obesity.

Adipocytes are capable of transdifferentiation from white adipocytes to beige or brown adipocytes, which have a higher thermogenic capacity and lipids stored in their vacuoles serve primarily as fuel for oxidative phosphorylation and heat production [24]. In this process, uncoupling protein UCP1 plays an important role by inducing a non-shivering thermogenesis in beige and brown adipocytes [44]. The *Ucp1* expression is positively regulated by interaction of a transcriptional cofactor for mitochondrial biogenesis PGC-1-α with PPARγ [45]. We hypothesized that due to the influence of QCT administration, transdifferentiation of adipocytes could occur in the white adipose tissue of ZDF rats. However, in ScWAT, Ucp1 mRNA expression was below the level of detectability despite of a strong tendency for *Pgc1α* being increased in obese QCT-treated rats. In the rWAT of obese ZDF rats, the expression of *Ucp1* was not affected by QCT. Nevertheless, another study in mice that received a high-fat diet showed a stimulatory effect of QCT administration on *Ucp1* expression in inguinal adipose tissue [46]. Since other genes are also involved in the transdifferentiation of white adipocytes, we measured the expression of markers of brown (*Cidea*), beige (*Tnfrsf9*) and white (*Hoxc9*) adipocytes. In the expression of all three markers, we noticed different pattern in the subcutaneous and visceral depots after QCT administration. While in ScWAT the expressions did not change, in rWAT they were reduced by QCT in obese rats. This effect may be attributed to the anti-adipogenic effect of QCT, when adipogenic differentiation of all three types of adipocytes (white, beige and brown) is inhibited.

One of the limitations of all studies with QCT is comparison of the QCT effects in different experiments due to its mode of administration to animals. Different ways of administration were employed. Besides using biscuit as a vehicle the QCT was administrated also by gavage as dispersed in water [47] or 1% methyl cellulose solution [33]. Regarding concentration range it was from 10 to 100 mg/kg/day. Taking in account low solubility of QCT in water, different routes of drug administration give distinct physiological effects even at the same concentration used. In addition, we do not know the metabolic fate of QCT in lean and obese animals. We might suggest that in obese rats, QCT is more stored in fat tissue. So, we might speculate that QCT is more effective in obese rat adipose tissue.

Anti-diabetic and insulin-sensitizing effects of QCT have been recently summarized very well in a study by Zhou et al. [48]. In animals, QCT can promote insulin secretion, decrease insulin resistance, inhibit inflammation and oxidative stress, and reduce liver fat accumulation. However, administration of QCT has mainly been investigated in models of diet-induced obesity or insulin resistance, or in models of streptozotocin-induced diabetes. The metabolic effect of QCT was determined mainly in the liver, pancreas and skeletal muscle. Stewart’s study even showed the inability of dietary QCT to ameliorate insulin resistance in the liver that developed after short-term consumption of a high-fat diet in mice [49]. QCT administered to obese Zucker rats in dose 10 mg/kg/day orally by gavage for 10 weeks improved dislipidemia, hyperinsulinemia and hypertension [33]. However, these changes could be at least in part attributed to a reduction in body weight gain despite the same food intake compared to obese controls. The ZDF model, used in our study, represents a genetic model of obesity with developed insulin resistance and diabetes and we focused mainly on adipose tissue. The results from our study in animals with fully developed diabetes did not confirm the anti-diabetic and insulin-sensitizing effect of QCT, and therefore we believe that preventive treatment would be more effective. Moreover, some functional studies such as measurement of activity of lipolytic enzymes, glucose transport into adipocytes or glucose tolerance test would contribute to better knowledge and interpretation of presented data.

## Conclusions

In conclusion, QCT reduces adipocyte size in obese diabetic ZDF rats in ScWAT and activated lipolytic and inhibited adipogenic pathways are probably involved. As a result, an increased release of FFAs into the circulation occured. However, further research is needed to fully describe these mechanisms. Our results showed that the reduction of adipocytes is accompanied by a decrease in the expression of inflammatory markers in ScWAT in vivo. In the group of obese ZDF rats, there is also a decrease in the expression of ROS-forming enzymes. Our results indicate improving effect of QCT on redox homeostasis and inflammation in ScWAT and our study expands knowledge about the metabolic effects of flavonoids in vivo and points out that inhibition of adipogenesis may not always lead to improved metabolic health.

## Data Availability

No datasets were generated or analysed during the current study.
